# Sulphenylation of CypD at Cysteine 104: A Novel Mechanism by Which SO_2_ Inhibits Cardiomyocyte Apoptosis

**DOI:** 10.3389/fcell.2021.784799

**Published:** 2022-01-18

**Authors:** Boyang Lv, Hanlin Peng, Bingquan Qiu, Lulu Zhang, Mei Ge, Dingfang Bu, Kun Li, Xiaoqi Yu, Jiantong Du, Liu Yang, Chaoshu Tang, Yaqian Huang, Junbao Du, Hongfang Jin

**Affiliations:** ^1^ Department of Pediatrics, Peking University First Hospital, Beijing, China; ^2^ Key Laboratory of Green Chemistry and Technology, Ministry of Education, College of Chemistry, Sichuan University, Chengdu, China; ^3^ Department of Ophthalmology, Peking University First Hospital, Beijing, China; ^4^ Key Laboratory of Molecular Cardiology, Ministry of Education, Beijing, China; ^5^ Department of Physiology and Pathophysiology, Peking University Health Science Centre, Beijing, China

**Keywords:** sulfur dioxide, cardiomyocyte, apoptosis, sulphenylation, CypD

## Abstract

**Objectives:** The study was designed to explore the role of endogenous gaseous signaling molecule sulfur dioxide (SO_2_) in the control of cardiomyocyte apoptosis and its molecular mechanisms.

**Methods:** Neonatal mouse cardiac myocytes (NMCMs) and H9c2 cells were used in the cell experiments. The endogenous SO_2_ pathway including SO_2_ level and the expression of SO_2_-generating enzyme aspartate aminotransferase 1/2 (AAT1/2) were detected in NMCMs. The apoptosis of cardiomyocytes was examined by a TUNEL assay. The cleavage and the activity of apoptotic proteins caspase9 and caspase3 were measured. The content of ATP, the opening of mitochondrial permeability transition pore (mPTP), and the cytochrome c (cytc) leakage were detected by immunofluorescence. The sulphenylation of cyclophilin-D (CypD) was detected by biotin switch analysis. The four CypD mutant plasmids in which cysteine sites were mutated to serine were constructed to identify the SO_2_-affected site *in vitro*.

**Results:** ISO down-regulated the endogenous SO_2_/AAT pathway of cardiomyocytes in association with a significant increase in cardiomyocyte apoptosis, demonstrated by the increases in apoptosis, cleaved-caspase3/caspase3 ratio, and caspase3 activity. Furthermore, ISO significantly reduced ATP production in H9c2 cells, but the supplement of SO_2_ significantly restored the content of ATP. ISO stimulated mPTP opening, resulting in an increase in the release of cytc, which further increased the ratio of cleaved caspase9/caspase9 and enhanced the protein activity of caspase9. While, the supplementation of SO_2_ reversed the above effects. Mechanistically, SO_2_ did not affect CypD protein expression, but sulphenylated CypD and inhibited mPTP opening, resulting in an inhibition of cardiomyocyte apoptosis. The C104S mutation in CypD abolished SO_2_-induced sulphenylation of CypD, and thereby blocked the inhibitory effect of SO_2_ on the mPTP opening and cardiomyocyte apoptosis.

**Conclusion:** Endogenous SO_2_ sulphenylated CypD at Cys104 to inhibit mPTP opening, and thus protected against cardiomyocyte apoptosis.

## 1 Introduction

Myocardial injury is an important pathophysiological process in a variety of cardiovascular diseases (Jin et al., 2013), including hypertension, heart failure, and coronary heart disease (Leong et al., 2017). Cardiomyocyte apoptosis plays a crucial part in cardiovascular diseases (Singh et al., 2011). Therefore, clarifying the mechanisms underlying cardiomyocyte apoptosis in cardiovascular diseases has always been the focus of the research (Abbate and Narula, 2012). However, the exact mechanisms for cardiomyocyte apoptosis have not yet been fully clarified.

Sulfur dioxide (SO_2_) had been regarded as a waste gas in air pollution. Recent literature has shown that cardiovascular tissues can produce SO_2_ endogenously (Du et al., 2008a). Increasing evidences have confirmed that the endogenous SO_2_ exerts crucial cardiovascular pathophysiologic functions (Zhou et al., 2020). Our previous animal study showed that the down-regulated endogenous SO_2_/AAT pathway might be involved in the mechanisms underlying isoproterenol (ISO)- induced myocardial damage, and the protective role of SO_2_ might be related to the enhancement of myocardial antioxidant capacity in rats (Liang et al., 2011). In addition, Jin et al. (2013) found that myocardial injury is related to the inhibition of the bcl2/cytc/caspase9/caspase3 pathway mediated by SO_2_. However, the molecular mechanisms by which endogenous SO_2_ protected cardiomyocyte injury and apoptosis are completely not clear.

Mitochondrial permeability transition refers to the process that the inner membrane of mitochondria allows solutes up to 1.5 kDa to pass freely (Kwong and Molkentin, 2015). Prolonged opening of mitochondrial permeability transition pore (mPTP) can lead to mitochondrial energy dysfunction, swelling, rupture, apoptosis, and necrotic cell death (Zhou et al., 2019). Cyclophilin-D (CypD) locates in the mitochondrial matrix and is a peptidyl-prolyl isomerase. CypD translocates from mitochondrial matrix to mitochondrial inner membrane and binds to adenine nucleotide translocator (ANT) to initiate the formation of mPTP complex. Therefore, CypD acts as an important regulator of switching the mPTP opening (Sun et al., 2019). It has been reported that a variety of post-translational modifications occur in CypD (Elrod and Molkentin, 2013), such as S-nitrosylation (Nguyen et al., 2011), acetylation (Amanakis et al., 2021), and phosphorylation (Hurst et al., 2020). As a small gaseous signaling molecule, SO_2_ regulates downstream proteins and exerts biological effects through post-translational sulphenylation. As a reversible form of post-translational modification, sulphenylation can oxidize cysteine mercaptan to sulphenic acid (Cys-SOH) and regulate protein function (Song et al., 2020). According to the amino acid sequence analysis of the CypD, the human CypD protein contains four cysteines. However, it is not clear whether endogenous SO_2_ can inhibit the opening of mPTP to reduce cardiomyocyte apoptosis through the sulphenylation of CypD.

Therefore, in this study, the role of the endogenous SO_2_ in the development of ISO-induced cardiomyocyte apoptosis was investigated. Moreover, we explored its mechanism from a new point of view that SO_2_ likely inhibits the opening of mPTP by chemically modifying CypD, thus alleviating cardiomyocyte apoptosis.

## 2 Materials and Methods

### 2.1 Reagent

Sodium hydrogen sulfite and sodium sulfite (NaHSO_3_/Na_2_SO_3_, freshly mixed at 1:3 M ratio, pH 7.4) were used as SO_2_ donors and purchased from Sigma (Zhang et al., 2021). Isoprenaline hydrochloride was purchased from Sigma (I5627). A chemically selective fluorescent probe SS-1 was provided by Professor Kun Li and Xiaoqi Yu. DAz-2 was used as a protein sulphenylation probe (13382, Cayman, Michigan, USA) to capture and enrich the sulphenylated protein. The primary antibodies in the present study included CypD (abcam, USA), AAT1 (Sigma, USA), AAT2 (Sigma, USA), β-actin (Zsbio, China), caspase9 (CST, USA), caspase3 (Beyotime, China), cleaved caspase3 (Beyotime, China), cytc (santa, USA), His (Zsbio, China), and β-tubulin (Zsbio, China). Human CypD wild type (WT), and C104S, C82S, C157S, and C203S mutant plasmids were constructed by Sangon Biotech. The information of three kinds of ATP plasmids used in this study are as follows: EcAT3.10 (Conley et al., 2017) was deposited at Addgene by Mathew Tantama (Addgene plasmid #107215); pm-iATPSnFR1.1 (Lobas et al., 2019) (Addgene plasmid #102549) and cyto-iATPSnFR1.0 (Lobas et al., 2019) (Addgene plasmid #102550) were deposited at Addgene by Baljit Khakh.

### 2.2 Cell Culture and Treatment

Culture of NMCMs: The kit for isolation of primary mouse cardiomyocytes was purchased from Thermo Fisher (Waltham, USA). In brief, the ventricular tissue parts from 1–2 days old C57BL/6J mouse neonates were subjected to modified enzymatic digestion. The enriched cardiomyocytes were cultured in DMEM with 1% penicillin-streptomycin solution (PS) and 10% fetal bovine serum (FBS). A cardiomyocyte growth supplement was added to inhibit the growth of the remaining fibroblasts.

NMCMs were divided randomly into control, ISO, ISO + SO_2_, and SO_2_ groups. For the control group, the cells were treated with the equivalent amount of saline for 48 h. For the ISO group, they were treated with 10 μmol/L ISO for 48 h. For the ISO + SO_2_ group, the cells were given 100 μmol/L SO_2_ donor for 30 min and 10 μmol/L ISO for 48 h (Ding et al., 2005). For the SO_2_ group, cells were treated with 100 μmol/L SO_2_ donor for 0.5 h and the equivalent amount of saline for 48 h.

H9c2 cell culture: H9c2 rat embryonic cardiomyocytes were purchased from the National Infrastructure of Cell Line Resource (China) and they were cultured in DMEM medium containing 10% FBS, 1% PS, and 1% glutathione in 5% CO_2_ at 37°C. After a 6 h of synchronous treatment in serum-free medium, the cell treatment and grouping were similar to those in NMCMs, except that in H9c2 cells the ISO treatment was 200 μmol/L for 24 h (Deng et al., 2017).

### 2.3 *In situ* Fluorescent Imaging of SO_2_

SS-1 is a chemically selective fluorescent probe for *in situ* visualization of SO_2_, developed and presented by Kun Li from Sichuan University, Sichuan, China (Yang et al., 2017). As mentioned in the previous study, SO_2_ in NMCMs was tested with an SS-1 probe (Huang et al., 2021a). The cells were incubated with a 10 μM probe for 30 min at 37°C and then they were washed with PBS for removing the unlabeled probe. Subsequently, the cells were fixed with 4% paraformaldehyde solution. Finally, DAPI was added to stain nuclei. The green fluorescence detected by Olympus confocal laser scanning microscope (CK40, Olympus, Japan) is considered to be a positive signal.

### 2.4 AAT Activity Detected by Colorimetric Assay

AAT assay kit (Njjcbio, Nanjing, China) was used as described previously (Zhang et al., 2021). Firstly, cells were collected and lysed on ice with pre-cooled PBS for 30 min. The substrate solution and tested samples were added into the 96-well plate, and reacted at 37°C for 30 min. Then, 2,4-dinitrophenylhydrazine was added to each well, and the plate was placed at 37°C for 20 min. Finally, sodium hydroxide solution (200 μl, 0.4 mol/L) was added and gently mixed into each well. The plate was incubated at room temperature for 15 min. Finally, the optical density was measured at the absorption wavelength of 510 nm.

### 2.5 *In situ* Detection of Apoptosis in NMCMs and H9c2 Cells by TdT-Mediated dUTP Nick End Labeling Assay

*In situ* cell death detection kit (Roche, Mannheim, Germany) was used as described previously (Du et al., 2018). Firstly, after gently rinsed with PBS, the cells were fixed in 4% paraformaldehyde for 1 h. Subsequently, they were permeabilized in 0.3% triton-X100 solution for 2 min. They were incubated with the TUNEL reaction mixture for 60 min at 37°C, preventing from light after washing with PBS. Finally, the nuclei were stained by a DAPI dye. The fluorescence images were captured with the help of a confocal laser scanning microscope (CK40, Olympus, Japan). The cell apoptosis was measured by using the percentage of TUNEL positive cells to the total DAPI positive cells (Du et al., 2018).

### 2.6 ATP Fluorescence Intensity of Cytoplasm and Cell Surface Monitored by Immunofluorescence Method in H9c2 Cells

H9c2 cells were seeded and grown to 50% confluency, and then pmiATPSnFR1.1 and cyto-iATPSnFR1.0 were respectively transfected into H9c2 cells, which were replaced with a complete culture medium after a 6 h-transfection. The cells were imaged under a confocal laser scanning microscope (TCS SP8, Leica, Wetzlar, Germany) by the excitation at 473 nm and the emission at 525 nm.

### 2.7 ATP Fluorescence Intensity of Extracellular Matrix Monitored by Immunofluorescence Method in H9c2 Cells

The plasmid ecAT3.10 was transfected into 50% confluent H9c2 cells with a lipofectamine 3000 transfection reagent (Invitrogen, Carlsbad, CA, United States). After a 6 h-transfection, the freshly completed culture medium was replaced. The activity of the ecATeam sensor was measured by examining the fluorescence intensities in CFP, CFP-YFPFRET, and YFP channels as described in the previous study. The settings of the bandpass filter were identical to those previously reported (Conley et al., 2017). The ratio of CFP-YFPFRET to YFP is expressed as the activity of the ecATeam biosensor. The conformational change of the probe induced by ATP binding to the ecATeam probe increases the Förster resonance energy transfer (FRET) between the CFP donor and the YFP receptor (Imamura et al., 2009). Therefore, the higher the ratio of CFP-YFPFRET to CFP, the more extracellular ATP binding, which indirectly reflects the extracellular ATP content.

### 2.8 Detection of Enzymatic Activities of Caspase3 and Caspase9 in H9c2 Cells

The enzymatic activities of caspase3 and caspase9 in H9c2 cells were detected with the commercial caspase3 and caspase9 activity kit, respectively (Applygen, Beijing, China) (Wang X. et al., 2019). Briefly, with the lysis buffer, the cells were lysed for 30 min at 4°C, and then centrifuged at 12,000 g for 5 min to harvest the supernatant. The concentration of protein was measured by the Bradford method. Then, 50 μg of cell lysate mixed with a reaction reagent was added to each well in a 96-well plate in order and then incubated for a period of 2 h at 37°C away from light. The activity of caspase3 or caspase9 was calculated from the absorbance value at 405 nm.

### 2.9 Detection of the mPTP Opening in NMCMs and H9c2 Cells

The mPTP opening was detected using the mPTP detection kit (Genmed, Shanghai, China) according to the instruction of the manufacture. The detection principle is that the fluorescence of calcein-AM is quenched when it leaks from mitochondria via the opening mPTP. Therefore, the strong green fluorescence represents the closed mPTP while the faint green fluorescence represents the opening mPTP. After completing the cell experiment, the cells were rinsed and incubated with the staining working solution for 20 min at 37°C in the dark. The remaining staining solution was removed by twice rinse with the cleaning solution. After the fixation with 4% paraformaldehyde, the fluorescence was observed by using a laser confocal microscopy (CK40, Olympus, Japan) with 488 nm excitation (Ex) and 505 nm emission (Em) settings (Song et al., 2018).

### 2.10 Detection of the Leakage of Mitochondrial Cytc in NMCMs

To determine the cytc subcellular localization, NMCMs were firstly incubated with the pre-warmed medium which contained 200 nmol/L MitoTracker (Life Technologies, USA) for 1 h. After the fixation with 4% paraformaldehyde, the cells were permeabilized in 0.1% Triton-X100 solution for 30 min, and then the cells were incubated with the cytc primary antibody at 4°C overnight. After the triplicate rinses with PBS buffer, an Alexa 594-conjugated secondary antibody (Life Technologies, USA) was added and then incubated for 90 min at 37°C in a dark container. Finally, images were captured with laser confocal microscopy (SP8-STED, Leica, Germany) (Wang X. et al., 2019).

### 2.11 CypD Sulphenylation Detection by Biotin Switch Analysis

Sulphenylation of CypD in the H9c2 cells and purified CypD protein was detected by the BSA method as previously reported (Huang et al., 2021a). The H9c2 cells were lysed in a non-denaturing lysis buffer (Applygen, Beijing, China) containing 5 mM DAz-2 for 20 min. The supernatant was collected by centrifuging at 16,000 g for 4 min at 4°C and gently shaken for 2.5 h at 37°C to label the DAZ-2. The DAz-2-labelled samples reacted with 250 μM p-biotin in a water bath for 2 h at 37°C. Then, the hyperlinked Neutral Affinity Protein™ was added at a volume ratio of 1–10 (Thermo Fisher Science) and incubated in a shaker for 4 h to capture the sulfenylated protein at 4°C. The sulphenylated proteins were mixed with a non-denaturing sample buffer and boiled for 10 min. Then, the supernatant was collected by centrifuging at 5,000 g for 10 min and it was subjected to western blot for the sulphenylation of CypD.

The human purified CypD protein (Abnova) was randomly divided into 3 groups: control, SO_2_, and SO_2_+DTT groups. The amount of purified protein used in each group was 0.1 μg. The protein was incubated for 2 h at 37°C. After the termination of the incubation experiment, the purified protein was divided into two portions to detect the sulphenylation of CypD and total CypD, respectively. The CypD sulphenylation was detected according to the abovementioned protocol in the cell experiment.

### 2.12 *In situ* Detection of CypD Sulphenylation in H9c2 Cells

A sulphenylated protein cell-based detection kit (Cayman, USA) with a DAz-2-based fluorescence probe was used to visualize the sulphenylated proteins in cells (Song et al., 2020). The cells were treated with 100 μM SO_2_ for 1 h or SO_2_ plus 200 μM DTT for 15 min. The sulphenylation of CypD in H9c2 cells was observed by the co-localization of the fluorescent signals indicating CypD and sulphenylated proteins. Nuclei were stained with DAPI dye. The sulphenylated protein, CypD protein, and nuclei exhibited green, red, and blue fluorescence, respectively, with confocal laser-scanning microscope (Leica, Germany).

### 2.13 CypD Plasmid Transfection

The pcDNA3.1 vector for human CypD-WT, CypD-C82S, CypD-C104S, CypD-C157S, and CypD-C203S mutant plasmids was constructed by Sangon Biotech (Shanghai, China). When H9c2 cells grew to approximately 50–60% confluence, the cells were treated with jetPEI TM reagent (Polyplus-transfection, France) before treatment.

### 2.14 Western Blotting Analysis

The protein expression of AAT1, AAT2, cleaved caspase3, caspase3, cleaved caspase9, caspase9, CypD, and His in NMCMs and H9c2 cells was detected by a standard western blotting analysis as described in the previous study (Huang, et al., 2021a). Briefly, the cells were lysed using protein lysis buffer for 20 min at 4°C. The total protein was harvested and quantitated by the BCA method. The proteins were subjected to electrophoresis and they were subsequently transferred to nitrocellulose membranes. The protein bands were treated with primary antibodies at 4°C overnight, respectively. Then, at room temperature, they were incubated with the corresponding horseradish peroxidase-coupled secondary antibody for 1 h. Finally, the protein bands were incubated by using the enhanced chemiluminescent western blotting substrate kit (GE, Pittsburgh, PA, United States) in a FluorChem M MultiFluor System (Protein Simple, San Francisco, CA, United States).

### 2.15 Gene Ontology Cellular Component Analysis

A dataset containing a total of 658 SO_2_-mediated sulfenylated vascular smooth muscle cell (VSMC) proteins was from Huang’s research (Huang et al., 2021a). The gene ontology cellular component analysis of the 658 SO_2_-mediated sulfenylated VSMC proteins was performed by g: Profiler (https://biit.cs.ut.ee/gprofiler) (version e104_eg51_p15_3922dba). The parameters were set as follows: organism (Rattus norvegicus); statistical domain scope (only annotated genes); significance threshold (g:SCS threshold); and *p* values <0.05 indicated statistical significance.

### 2.16 Statistical Analysis

The statistical analysis was conducted using SPSS 17.0 (IBM, USA) and Graphpad Prism 8.0 (GraphPad Software Inc., San Diego, CA, United States). The data are expressed as mean ± standard error. A comparison among multiple groups was performed by ANOVA followed by LSD post-doc analysis if the data were normally distributed, while followed by Dunnett T3 post-doc analysis if the data were not normally distributed. A statistical significance is set by *p* < 0.05.

## 3 Results

### 3.1 Endogenous SO_2_ Controls ISO-Induced Apoptosis of Cardiomyocytes

**FIGURE 1:**
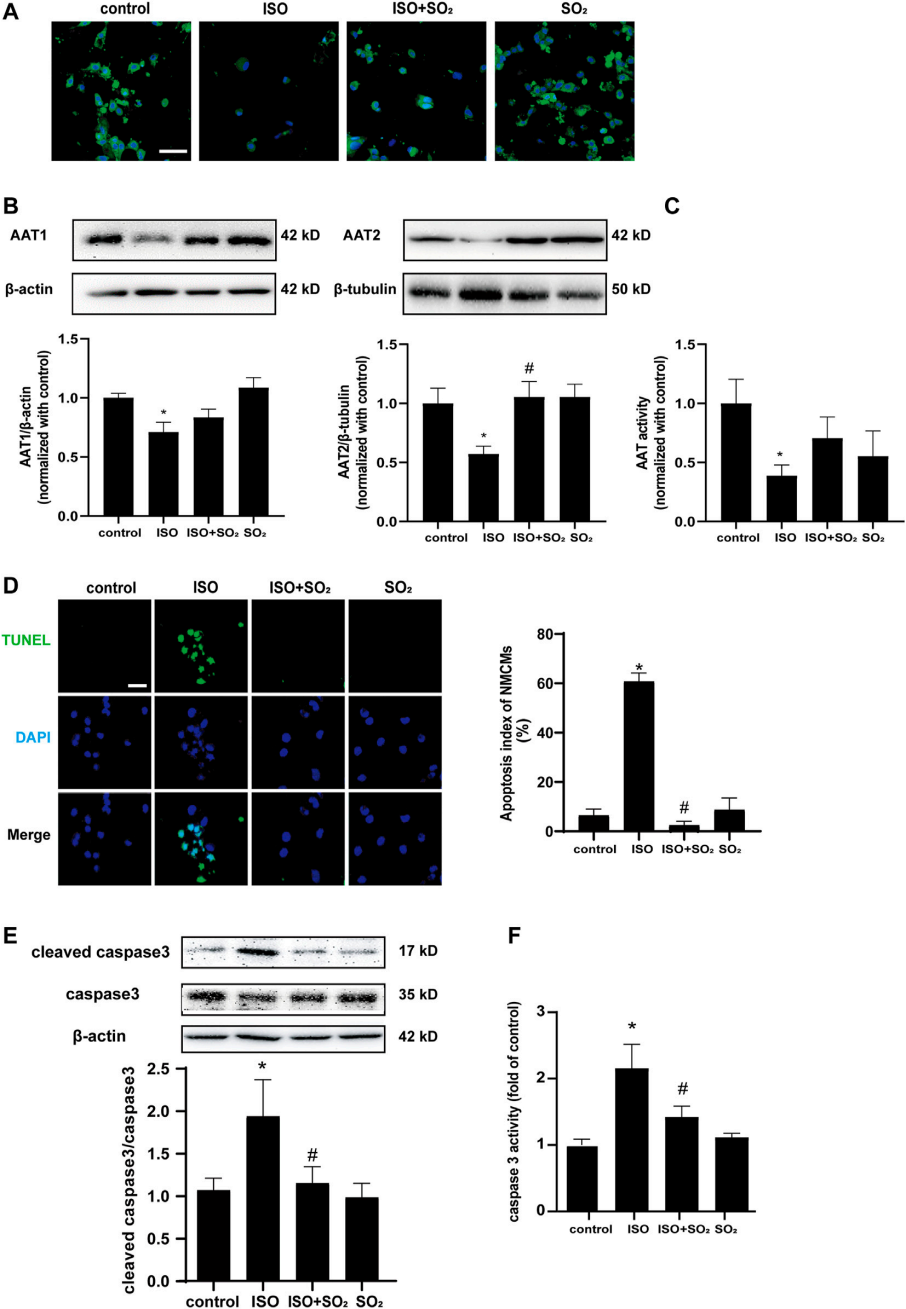
Endogenous SO_2_ controls ISO-induced apoptosis of cardiomyocytes. **(A)** SO_2_ production in NMCMs was tested with *in situ* fluorescent SO_2_ probe (green color, magnification, ×600; scale bar: 40 μm). **(B)** AAT1 and AAT2 expressions in NMCMs were measured by western blot. **(C)** AAT activity in NMCMs was detected by colorimetric assay. **(D)** The apoptosis of NMCMs was tested by TdT-mediated dUTP nick end labeling (TUNEL) assays (magnification, ×600; scale bar: 40 μm). **(E)** The caspase3 cleavage in H9c2 cells was measured by using western blot method. **(F)** A quantitative caspase3 activity analysis was done by the colorimetric kit. Data are expressed as mean ± SEM. ∗*p* < 0.05 versus control group; ^#^ *p* < 0.05 versus ISO group.

ISO-stimulated NMCMs showed a marked decrease in SO_2_ content as compared with the control group as evidenced by a significant decrease in SO_2_-specific green fluorescence, and an obvious decrease in AAT1 and AAT2 protein expression, and AAT activity in the ISO-stimulated NMCMs (Figures 1A–C), accompanied by a significant increase in cell apoptosis, as evidenced by an increase in apoptotic cells, the cleaved-caspase3/caspase3 ratio, and caspase3 activity demonstrated by colorimetric assay (Figures 1D–F). The supplementation of SO_2_ donors in ISO-stimulated NMCMs restored SO_2_ content while significantly reduced the apoptotic cells, lowered the cleaved-caspase3/caspase3 ratio, and inhibited caspase3 activity (Figure 1). Therefore, the abovementioned data suggested that ISO stimulation downregulated the endogenous SO_2_/AAT pathway and promoted cardiomyocyte apoptosis; whereas adequate endogenous SO_2_ inhibited the apoptosis of cardiomyocytes.

### 3.2 SO_2_ Blocked ISO-Induced mPTP Opening and Subsequent Cardiomyocyte Apoptosis

**FIGURE 2:**
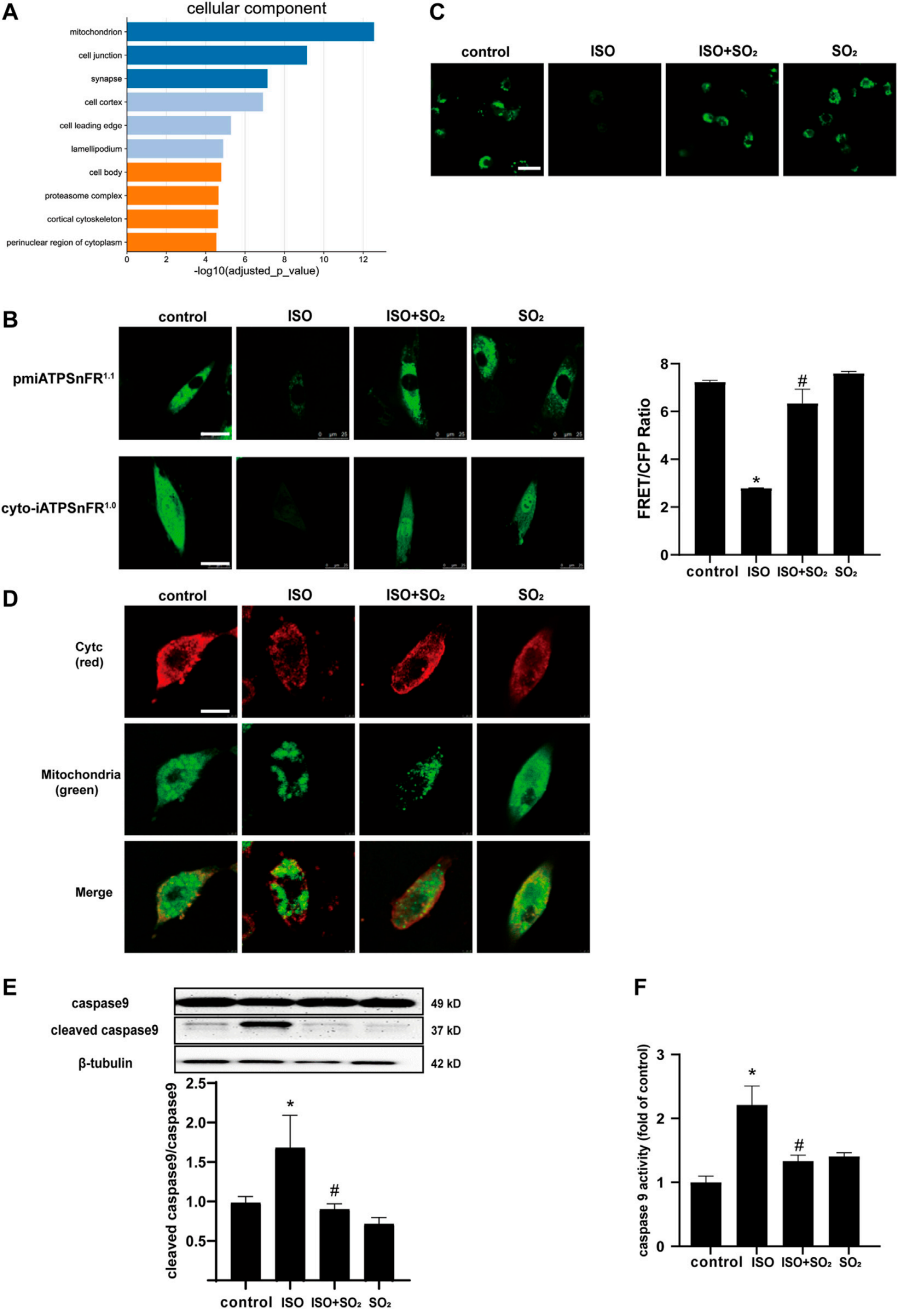
SO_2_ blocked ISO-induced mPTP opening and subsequent cardiomyocyte apoptosis. **(A)** GO-enriched cellular component (CC) reanalysis of the SO_2_-affected redox proteomic dataset. **(B)** The fluorescence intensity of cell surface and cytoplasmic ATP were investigated by the transfection of H9c2 cells with pmiATPSnFR^1.1^ and cyto-iATPSnFR^1.0^, respectively (magnification, ×600; scale bar: 25 μm). The ATP fluorescence intensity of extracellular matrix monitored by immunofluorescence method in H9c2 cells. **(C)** The mPTP opening in H9c2 cells was tested with calcein-AM. The green fluorescence quenching indicated the opening of mPTP (magnification, ×600; scale bar: 20 μm). **(D)** The cytochrome c (cytc) leakage from the mitochondria was tested by using immunofluorescence microscopy, with red fluorescence representing cytc and green fluorescence representing mitochondria (magnification, ×600; scale bar: 50 μm). **(E)** Caspase9 cleavage measured by western blotting. **(F)** A quantitative caspase9 activity analysis was done by using a commercial colorimetric kit. Data are expressed as mean ± SEM. ∗*p* < 0.05 versus control group; ^#^ *p* < 0.05 versus ISO group.

We performed a GO-enriched cellular component (CC) reanalysis of the SO_2_-affected redox proteomic dataset and showed that differential proteins regulated by SO_2_ were significantly enriched in mitochondria (*p* = 2.829 × 10^–13^) (Figure 2A). Furthermore, we compared the ATP fluorescence intensity in the cytoplasm, membrane, and extracellular matrix of H9c2 cells and found that ATP production was significantly reduced in H9c2 cells in the ISO group in comparison with the control group, and the supplementation with SO_2_ significantly restored the ATP content (Figures 2B,C), suggesting that mitochondria might be an important target for the cytoprotective effect of SO_2_ on cardiomyocytes.

The mPTP opening is an important event in the development of cardiomyocyte apoptosis under a variety of cardiac pathological conditions (Ahmad et al., 2019). Therefore, the present study analyzed the mitochondrial mPTP opening and downstream events. The results showed that ISO-stimulated NMCMs showed an increased mitochondrial mPTP opening, an increased cytc leakage from mitochondria, and an enhanced cleaved caspase9/caspase9 ratio and caspase9 activity in comparison with the control group; while SO_2_ inhibited ISO-induced mPTP opening, suppressed the cytc release and reduced the ratio of cleaved caspase9/caspase9, thereby inhibiting apoptosis (Figures 2D–F). The results suggested that SO_2_ significantly blocked ISO-opened mPTP, which might be involved in the mechanisms by which SO_2_ inhibited cardiomyocyte apoptosis.

### 3.3 SO_2_ Sulphenylated CypD and Thereby Blocked the mPTP Opening and Cardiomyocyte Apoptosis

CypD, a peptidyl-prolylcis-trans isomerase (PPI) present in the mitochondrial matrix, is an important redox-regulation-dependent mPTP regulator (Lam et al., 2015). Therefore, we tested if CypD was a candidate target of SO_2_ to explore the possible mechanism by which SO_2_ blocked mitochondrial mPTP opening.

**FIGURE 3:**
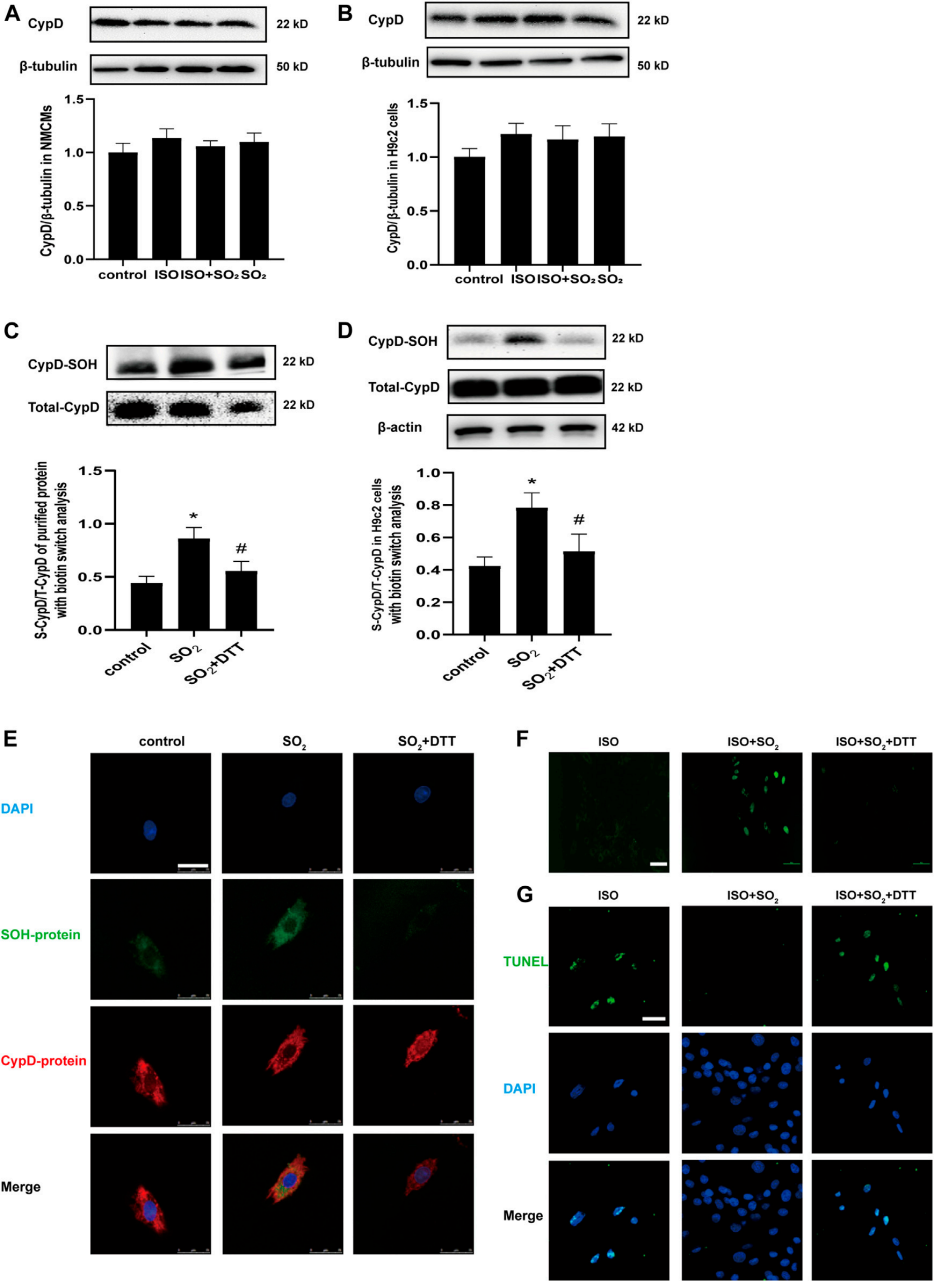
SO_2_ sulphenylated CypD and thereby blocked mitochondrial mPTP opening and apoptosis. CypD expressions in NMCMs and H9c2 cells were measured by western blot in **(A)** and **(B)**, respectively. **(C)** Sulphenylation of CypD in the purified protein with biotin switch analysis. **(D)** Sulphenylation of CypD in H9c2 cells with biotin switch analysis. **(E)** The co-localization of sulphenylated protein and CypD in H9c2 cells as detected with a DAz-2-based fluorescent probe and CypD antibody under a confocal laser-scanning microscope (magnification, ×600; scale bar: 25 μm). **(F)** The mPTP opening in H9c2 cells was detected with calcein-AM (magnification, ×400; scale bar: 40 μm). **(G)** The apoptosis of H9c2 cells was tested by using the TdT-mediated dUTP nick end labeling (TUNEL) method (magnification, ×600; scale bar: 25 μm).

Firstly, there were no significant effects of SO_2_ on the protein level of CypD either in NMCMs or in H9c2 cells as shown in Figures 3A,B (*p* > 0.05). However, the sulphenylation of CypD in human purified CypD protein in the SO_2_ group was markedly higher than that of the control group, which was blocked by a sulfhydryl reducing agent DTT treatment (*p* all<0.05) (Figure 3C).

Furthermore, the quantitative analysis and *in situ* visualization of CypD sulphenylation in the H9c2 cells were performed. The results revealed that in comparison with the control, the sulphenylation of CypD was increased in H9c2 cells of the SO_2_ group, which was also successfully blocked by DTT (*p* all<0.05) (Figure 3D). The multi-color confocal images showed that the co-localization of the fluorescent signals indicating CypD and sulphenylated proteins was strong in H9c2 cells of the SO_2_ group but weak in that of the control group and SO_2_+DTT group (Figure 3E). Correspondingly, the mPTP opening and cell apoptosis were inhibited in the cells of the ISO + SO_2_ group in comparison with the ISO group, while DTT treatment reversed the above protective effect of SO_2_ (Figures 3F,H).

The above facts indicate that SO_2_ can directly sulphenylate CypD protein, which might be associated with SO_2_-inhibited mPTP opening and cell apoptosis.

### 3.4 The CypD Cys104 Might Be a Novel Target for SO_2_ to Inhibit mPTP Opening and Cardiomyocyte Apoptosis

**FIGURE 4:**
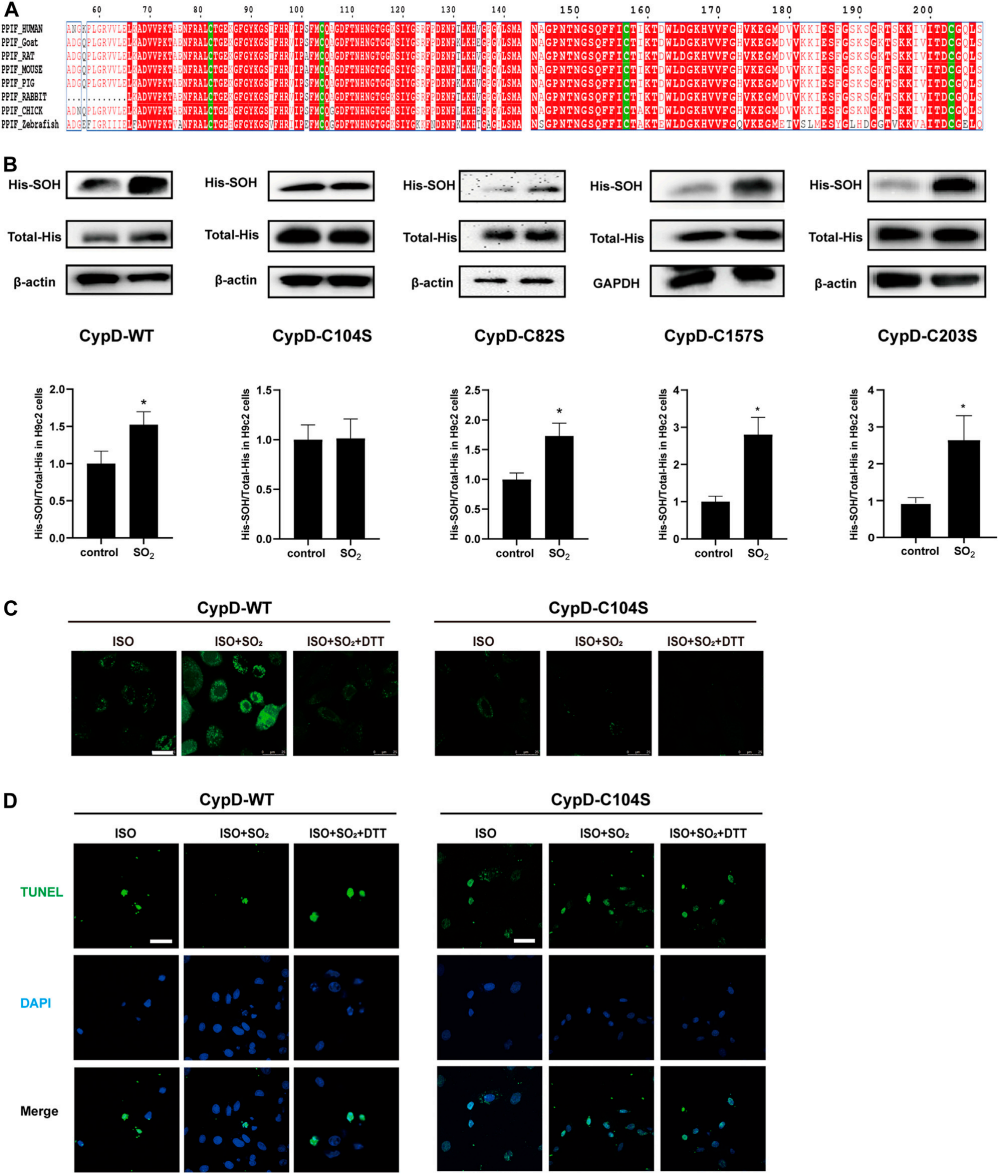
The CypD Cys104 might be a novel target for SO_2_ to inhibit cardiomyocyte apoptosis. **(A)** Analysis of sequence homology of CypD proteins from different species. The sequences were archived from the UniProt database. **(B)** Sulphenylation of CypD in H9c2 cells transfected with WT or C104S, C82S, C157S, C203S-mutated CypD plasmids was measured by biotin switch analysis. **(C)** The mPTP opening in H9c2 cells transfected with WT or C104S-mutated CypD plasmid measured by the mPTP detection kit (magnification, ×600; scale bar: 25 μm). **(D)** The apoptosis in H9c2 cells transfected with WT or C104S-mutated CypD plasmid measured by using the TdT-mediated dUTP nick end labeling (TUNEL) method (magnification, ×600; scale bar: 40 μm).

The human purified CypD protein has four cysteine sites: Cys82, Cys104, Cys157, and Cys203 (Linard et al., 2009). Homology analysis of CypD protein sequences among diverse species including human, goat, rat, mouse, pig, rabbit, chick and zebrafish showed that these four cysteine sites are all highly conserved among these different species (Figure 4A). Therefore, we mutated the four cysteine sites Cys82, Cys104, Cys157, and Cys203 to serine in the CypD protein, respectively, to clarify the precise site on which SO_2_ affected CypD and then inhibited the cardiomyocyte apoptosis. The sulphenylation site screening results showed that the sulphenylation of CypD-His could be induced by SO_2_ treatment in the H9c2 cells transfected with CypD-WT, C82S, C157S, and C203S plasmids, except in the cells transfected with CypD C104S plasmid (Figure 4B). The results suggested that the Cys104 in the CypD protein might be the sulphenylation site of SO_2_.

Furthermore, the mPTP opening and the apoptosis of H9c2 cells was examined to identify if the CypD Cys104 might be the target of SO_2_ affecting the cardiomyocyte apoptosis. The results showed that in the H9c2 cells transfected with CypD-WT plasmid, the mPTP opening in the ISO-stimulated cells was increased and the percentage of apoptotic cells was decreased by the SO_2_ treatment, while DTT could reverse the effect of SO_2_. In contrast, in the H9c2 cells transfected with CypD-C104S plasmid, SO_2_ treatment failed to affect the ISO-stimulated mPTP opening and the apoptosis (Figures 4C,D). The result further confirmed that SO_2_ sulphenylated CypD cysteine at Cys104, thereby inhibiting cardiomyocyte apoptosis.

## 4 Discussion

In the present study, we revealed, for the first time, a novel mechanism by which the endogenous SO_2_/AAT pathway controlled ISO-induced cardiomyocyte apoptosis, and the SO_2_-induced CypD sulphenylation inhibited the opening of mPTP, the downstream cytc leakage, and the activation of caspase9, thereby attenuating cardiomyocyte apoptosis. The sulphenylation of CypD cys104 is a key target for SO_2_ to inhibit apoptosis in cardiomyocytes.

Previously, SO_2_ was considered to be a toxic gas and environmental pollutant. In recent years, it is shown that the SO_2_ can be endogenously synthesized via an enzymatic reaction catalyzed by AAT in cardiovascular tissues (Huang et al., 2016). The half-life time of SO_2_ was about 5–10 min, demonstrated by the fact that serum SO_2_ level was decreased by 50% about 5–10 min after the intravenous injection of SO_2_ donor (Du et al., 2008b). It has features of low molecular weight, continuous production, and fast diffusion and has extensive biological action independent of the membrane receptors (Huang et al., 2016). Endogenous SO_2_ exerts important cardiovascular effects. For example, the reduction of endogenous SO_2_ promotes the proliferation and migration of cardiac fibroblasts (Zhang et al., 2018). SO_2_ inhibits the VSMC proliferation by activating Cl^−^/HCO_3^-^_ exchangers and acidifying cells (Wang Y. et al., 2019). Endogenous SO_2_ alleviates angiotensin II-induced myocardial hypertrophy and cardiomyocyte autophagy (Chen et al., 2016). It was reported that the downregulated endogenous SO_2_/AAT pathway might be involved in the possible mechanisms underlying the myocardial injury. Liang *et al* found that the *in vivo* protective effect of SO_2_ on the myocardial injury was related to the increased myocardial antioxidant capacity (Liang et al., 2011). Jin et al. (2013) found that SO_2_ attenuated myocardial injury, in association with the inhibition of myocardial apoptosis. However, the mechanisms by which SO_2_ protects cardiomyocytes against apoptosis have not yet been elucidated.

In this study, we showed that ISO treatment resulted in a downregulation of the endogenous SO_2_/AAT2 pathway in NMCMs, as evidenced by a significantly downregulated AAT2 expression and SO_2_ content in the ISO-stimulated cardiomyocytes. Simultaneously, cell apoptosis was evident. While the supplementation of SO_2_ donors in ISO-stimulated NMCMs restored SO_2_ content accompanied with a decreased cell apoptosis, cleaved-caspase3/caspase3 ratio, and caspase3 activity. These results demonstrate that endogenous SO_2_ significantly inhibits ISO-induced apoptosis in cardiomyocytes.

Up to now, the possible molecular mechanisms by which endogenous SO_2_ controls ISO-induced myocardial apoptosis have been unclear. A previous study suggested that SO_2_ could significantly affect signaling pathways associated with cellular energy metabolism in the VSMCs (Huang et al., 2021a). A cellular component reanalysis of gene ontology enrichment on the previously reported SO_2_-affected protein dataset was conducted in the present study. The data showed that mitochondrion is one of the important subcellular components in which SO_2_-affected proteins were highly enriched. Furthermore, considering that the mitochondrion acts as a cell energy mill, the change of ATP level affected by the ISO and/or SO_2_ treatment was investigated. As single-wavelength and genetically encoded fluorescent sensors, pmiATPSnFR1.1 and cyto-iATPSnFR1.0 were used for imaging cell surface and cytosolic ATP, respectively. The structure of pmiATPSnFR1.1 and cyto-iATPSnFR1.0 contains epsilon subunit F0F1-ATP synthase which binds to ATP. The conformation of these sensors changes and the fluorescence changes from dim to bright when bound to ATP (Lobas et al., 2019). Additionally, extracellular matrix ATP binding to ecAT3.10 induces a conformational change that increases FRET between the CFP donor and YFP acceptor (Conley et al., 2017). Therefore, the higher the ratio of CFP-YFPFRET to CFP, the more extracellular ATP binding, which indirectly reflects the extracellular ATP content. The results showed that ISO significantly reduced cytoplasmic ATP, cell surface ATP, and extracellular matrix ATP, while the supplementation with SO_2_ significantly reversed the reduced ATP fluorescence intensity in the H9c2 cells of the ISO group. Taken together, the abovementioned results suggested that SO_2_ might target the mitochondria to exert anti-apoptosis effects in cardiomyocytes.

The function of mitochondria is finely regulated by intrinsic factors, such as mPTP (Penna et al., 2013). Therefore, we, for the first time, examined the effect of SO_2_ on the opening of mPTP and its downstream events (cytc release, caspase9 activation, and apoptosis). The results showed that under ISO stimulation, the opening of mPTP was increased, the downstream leakage of cytc increased, the ratio of cleaved caspase9/caspase9 increased, and caspase9 activity was enhanced. While, SO_2_ supplementation reversed the above effects of ISO on the opening of mPTP and the downstream apoptosis, suggesting that SO_2_ might turn off mitochondrial mPTP and then inhibit cardiomyocyte apoptosis.

However, the possible mechanism by which SO_2_ blocks the mitochondrial mPTP opening has not yet been known. CypD is known to be the only protein identified to regulate the mPTP opening (Alam et al., 2015). CypD is a prolyl isomerase encoded by the Ppif gene and located within the mitochondrial matrix. Baines et al. (2005) found that the gene deletion of the Ppif in mice protected against ischemia/reperfusion-induced cell death, whereas the mice with CypD overexpression exhibited mitochondrial swelling and spontaneous cell death. Therefore, we firstly examined whether SO_2_ affected CypD protein expression in this study. The results revealed that SO_2_ did not impact the expression of CypD protein in the H9c2 cells with or without ISO stimulation. As discovered in the previous studies, the post-translational modifications might impact CypD activity (Nguyen et al., 2011; Sanchez et al., 2011; Hurst et al., 2020; Amanakis et al., 2021). For example, S-glutathionylation of CypD at the Cys203 prevented the binding between CypD and ANT, and therefore blocked mPTP (Nguyen et al., 2011; Sanchez et al., 2011). While, due to its oxidative capacity, SO_2_ was found to control the protein function by a sulfhydryl-dependent oxidative modification on the specific cysteinyl residue. Yao et al. found that SO_2_ promoted the disulfide-dependent dimerization of soluble guanylate cyclase to induce the vasodilate effect (Yao et al., 2016). More importantly, SO_2_ was reported to regulate the vascular function and structure by the sulphenylation, an important post-translational modification regulating protein function, on the target proteins such as AAT, Smad3 and NF-κB p65 (Chen et al., 2017; Song et al., 2020; Huang et al., 2021a). Sulphenylation refers to the oxidation of cysteine thiol groups (Cys-SH) to cysteine sulfenic acid (Cys-SOH) (Chen et al., 2017). Therefore, we explored whether CypD was able to be sulphenylated by SO_2_. Interestingly, the present cellular experiments and CypD purified protein assays demonstrated that SO_2_ could sulphenylate CypD protein to inhibit mPTP opening and cardiomyocyte apoptosis.

To further reveal the target site by which SO_2_ sulphenylated CypD and thereby inhibited cardiomyocyte apoptosis, we firstly performed the homology analysis of CypD protein sequences among diverse species and found that Cys82, Cys104, Cys157, and Cys203 are highly conserved. And then, the mutated CypD plasmids containing the site-directed mutant of C82S, C104S, C157S, and C203S were constructed and transfected into H9c2 cells, respectively. The screening results showed that the SO_2_ could sulfenylate the CypD in the H9c2 cells transfected with the mutant Cys82, Cys157, and Cys203, but, interestingly, SO_2_ failed to do so in the H9c2 cells transfected with the mutant CypD C104S. In accordance with the sulphenylation screening results, SO_2_ failed to prevent ISO-stimulated cell apoptosis in the H9c2 cells transfected with the mutant CypD C104S. These results further confirm that SO_2_ exerts an inhibitory effect on cardiomyocyte apoptosis by the sulphenylation of CypD at the Cys104.

As well known, the interaction among the different post-translational modifications on the same protein provided a fine mechanism for adjusting the protein function. For example, the persulfidation of p66Shc at Cys59 inhibited the phosphorylation at Ser36, and then blocked p66Shc activation to prevent H_2_O_2_-induced cellular senescence (Xie et al., 2014). In the oleic acid-induced A549 cell inflammatory experiment, endogenous SO_2_ sulphenylated NF-κB p65 in association with a decrease in the phosphorylation, nuclear translocation and DNA binding activity of NF-κB p65, suggesting that there might be interaction between the sulphenylation and phosphorylation of protein. While, it was reported that the phosphorylation of the Ser191 site of CypD leads to its binding to oligomycin sensitivity-conferring protein, which sensitizes mPTP opening (Hurst et al., 2020). Hence, the impact of the sulphenylation of CypD on the other post-translational modifications at other sites might also contribute to the sophisticated tuning of CypD and mPTP opening.

The mPTP opening and the loss of mitochondrial membrane potential (MMP) are two key elements involved in the cell apoptosis (Kroemer and Reed, 2000). Moreover, the opening of mPTP is closely related with the decrease in the MMP (Bossy-Wetzel et al., 1998; Hüser and Blatter, 1999; Joiner et al., 2012). Briefly, as a highly conductive and non-selective channel, the opening of mPTP increases the permeability of the inner mitochondrial membrane to ions and small solutes, and destroys the MMP and the proton gradient, resulting in the insufficient ATP production and cell apoptosis (Galluzzi et al., 2009; Kalani et al., 2018). Zhao et al. found that SO_2_ restored the destroyed MMP in the alveolar macrophages treated with the serum from rats of acute lung injury and prevented the cell apoptosis (Zhao et al., 2019). However, the effect of SO_2_-sulphenylated CypD on the MMP in the cardiomyocyte apoptosis is still unclear and merits further study.

## 5 Conclusion

**FIGURE 5:**
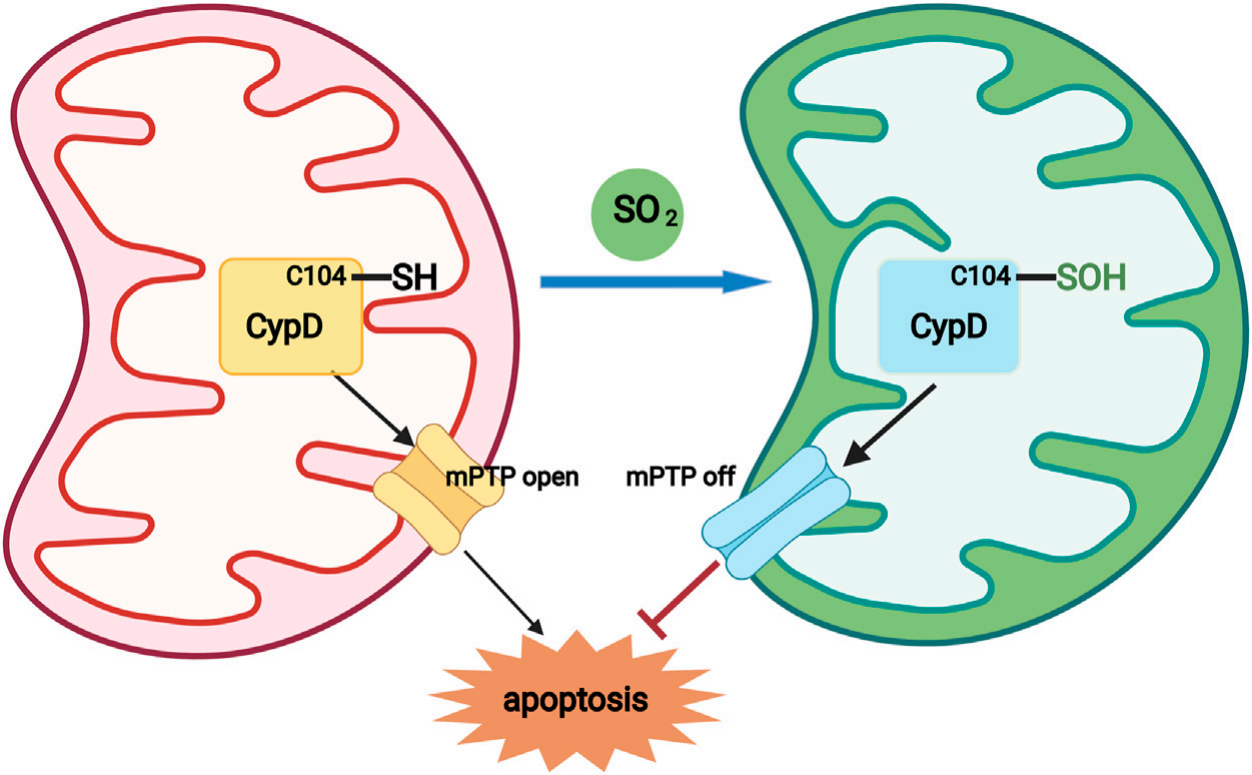
A diagram showing that a novel redox mechanism by which SO_2_ inhibits cardiomyocyte apoptosis. SO_2_ sulphenylated mitochondrial CypD at Cys 104, which acted as a switch-off to close the mPTP opening, thereby inhibiting mitochondria-dependent cardiomyocyte apoptosis.

Taken together, our data highlight the important protective role of SO_2_-dependent site-specific CypD S-sulphenylation in inhibiting mPTP opening and reducing apoptosis in cardiomyocytes (Figure 5). Along with the in-depth studies on the biological effect of endogenous SO_2_ and its mechanisms, the discovery of SO_2_-related prodrugs has become a hot topic for their potential therapeutic application (Day et al., 2016; Wang and Wang, 2018; Huang et al., 2021b). A variety of SO_2_ prodrugs based on the different releasing mechanisms, such as thiol-activated SO_2_ prodrug, thermally activated SO_2_ prodrug, hydrolysis-based SO_2_ prodrug, click reaction-based SO_2_ prodrug, and esterase-sensitive SO_2_ prodrug, developed to meet the different research requirement or clinical application in the future. Therefore, our results would not only deepen the understanding of the mechanisms by which endogenous SO_2_ inhibits myocardial apoptosis, but also provide new research ideas and potential therapeutic targets for myocardial protection in the future.

## Data Availability

All data supporting the findings of this study are available within this article or from the corresponding authors on request.
